# Intrathecal Humoral Immunity to Encephalitic RNA Viruses

**DOI:** 10.3390/v5020732

**Published:** 2013-02-15

**Authors:** Timothy W. Phares, Stephen A. Stohlman, Cornelia C. Bergmann

**Affiliations:** Departments of Neurosciences NC30, Lerner Research Institute, Cleveland Clinic Foundation, 9500 Euclid Avenue, Cleveland, OH 44195, USA; E-Mails: pharest@ccf.org (T.W.P.); stohlms2@ccf.org (S.A.S)

**Keywords:** antibody, antibody-secreting cell, B cell, central nervous system, humoral immunity, and RNA viruses

## Abstract

The nervous system is the target for acute encephalitic viral infections, as well as a reservoir for persisting viruses. Intrathecal antibody (Ab) synthesis is well documented in humans afflicted by infections associated with neurological complications, as well as the demyelinating disease, multiple sclerosis. This review focuses on the origin, recruitment, maintenance, and biological relevance of Ab-secreting cells (ASC) found in the central nervous system (CNS) following experimental neurotropic RNA virus infections. We will summarize evidence for a highly dynamic, evolving humoral response characterized by temporal alterations in B cell subsets, proliferation, and differentiation. Overall local Ab plays a beneficial role via complement-independent control of virus replication, although cross or self-reactive Ab to CNS antigens may contribute to immune-mediated pathogenesis during some infections. Importantly, protective Ab exert anti-viral activity not only by direct neutralization, but also by binding to cell surface-expressed viral glycoproteins. Ab engagement of viral glycoproteins blocks budding and mediates intracellular signaling leading to restored homeostatic and innate functions. The sustained Ab production by local ASC, as well as chemokines and cytokines associated with ASC recruitment and retention, are highlighted as critical components of immune control.

## 1. Introduction

The nervous system is a target for acute viral infections, as well as a reservoir of latent and persisting viruses [1,2,3,4,5,6,7]. In general, the absence of overt neurological deficits or pathology indicates effective immune control of persisting viruses in immunocompetent individuals. However, this balance is highly tenuous, as indicated by cases of JC virus-mediated progressive multifocal leukoencephalopathy (PML) in immunocompromised individuals with acquired immune deficiency syndrome or those receiving treatment for multiple sclerosis (MS) or lymphoma [3]. Similarly, the activation of herpes simplex virus (HSV) and cytomegalovirus in the nervous system can be devastating in immunocompromised individuals. Moreover, senescent immune responses in an increasingly aging population enhance disease susceptibility to both reactivating persistent viruses in the central nervous system (CNS), as well as to acute encephalitic arboviral infections. Numerous human infections involving the CNS, including those caused by measles, rubella, polio, varicella zoster, mumps, HSV and Japanese encephalitis virus (JEV), as well as lyme neuroborreliosis are characterized by intrathecal antibody (Ab) in the cerebral spinal fluid (CSF) consistent with the presence of local Ab-secreting cells (ASC) [8,9,10,11]. Although the causative agent still remains unknown in many cases of suspected viral encephalitis, detection of virus-specific immunoglobulin (Ig) in the CSF can be a reliable diagnostic tool to confirm a suspected viral encephalitis indicated by molecular analysis [12,13,14,15,16]. For example, acute poliomyelitis or encephalitis mediated by insect-borne viruses such as JEV are associated with virus-specific IgM and IgG in CSF within ~2 weeks of clinical presentation. While Ab persists over several months in the case of JEV [14], they appear more transient in poliomyelitis [15]. Overall, Ab detection may be more transient in cases of acute encephalitis, while it persists during chronic disease such as measles virus-associated subacute sclerosing panencephalitis [17,18]. A specific protective or detrimental role is often difficult to infer due to difficulties in obtaining longitudinal serum and CSF samples. Even when available, the role of serum *versus* intrathecal Ab cannot be readily distinguished. Overall, intrathecal humoral responses appear to be associated with protective rather than pathogenic functions. Thus, a beneficial outcome of JEV encephalitis is correlated with intrathecal IgG [19]. Similarly, intrathecal Ab synthesis may be an indicator of protection during CNS retrovirus infection [16,20,21]. Ab also correlates with reduced CNS viral load and milder clinical disease course in patients with tropical spastic paraparesis/HTLV-I-associated myelopathy [21]. An inverse correlation between intrathecal-neutralizing Ab and macrophage-tropic SIV was also observed in the SIV encephalitis model of HIV [20]. Lastly, the CSF of MS patients harbors Ab to multiple viruses prevalent in the Western population, e.g. varicella zoster, rubella, HSV-1 and JC viruses [2,13]. These Ab appear to be markers of MS and are not indicative of active disease due to virus infection [2]. Nevertheless, the potential danger of losing control of persisting CNS viruses became apparent by the development of PML following rituximab (anti-CD20 monoclonal Ab) reduction of circulating B cells during therapy for rheumatoid arthritis [22] and MS [23]. 

During experimental CNS infections, particularly by RNA viruses such as Sindbis, rabies and corona viruses, ASC play a vital local protective role [24,25,26,27,28,29,30,31]. The reliance on local ASC for sustained Ab output provides a potent complement-independent non-lytic mechanism of immune control within the CNS, potentially regulating a variety of neurotropic infections. Despite constituting a critical component controlling viral persistence, little is known about the origin and maintenance of ASC in the CNS or other specialized microenvironments. This review focuses on insights gained throughout the last decade on humoral immune responses within the CNS during encephalitis and persistent infections mediated by RNA viruses.

## 2. Development and Maintenance of B Cell Memory

**Figure 1:**
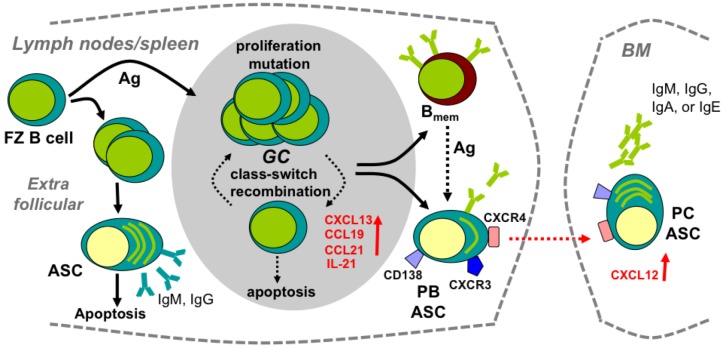
**Development of peripheral humoral responses.** Follicular zone (FZ) B cells in lymph nodes/spleen encounter antigen (Ag), proliferate and migrate to extrafollicular foci or lymphoid follicles. Extrafollicular B cells differentiate into short-lived antibody secreting cells (ASC), producing low-affinity Ab. B cells migrating to lymphoid follicles form germinal centers (GC) and differentiate into high Ab affinity plasma blasts (PB) and non-Ab-secreting memory B cells (B_mem_). ASC egress into the circulation and give rise to long-lived plasma cells (PC) residing primarily in bone marrow (BM). CXCR4–CXCL12 interactions mediate ASC homing to, and retention in, BM.

Following acute viral infection or immunization, antigen (Ag) in lymph nodes induces naive B cells to proliferate and migrate to extrafollicular foci or lymphoid follicles (Figure 1) [32,33]. Extrafollicular B cells, which differentiate into short-lived ASC, provide an early source of low-affinity Ab. By contrast, B cells migrating to lymphoid follicles in response to the CXCR5 ligand CXCL13, and CCR7 ligands CCL19 and CCL21, form germinal centers (GC) (Figure 1); these highly dynamic structures consist primarily of activated B cells, follicular dendritic cells (FDC) and T follicular helper (T_F_H) cells. FDC are radiation-resistant stromal cells that retain intact surface Ag and present Ag to the GC-invading B cells [34]. FDC further support GC formation by secreting CXCL13, a major chemoattractant of GC B cells and T_F_H cells, as well as IL-6 and B cell activating factor (BAFF), both of which promote GC reaction [34]. Interleukin (IL)-21 producing T_F_H cells constitute a T cell subset essential for GC maintenance and regulation of GC B cell clonal expansion and affinity maturation [35,36]. GC reactions lead to B cell differentiation into two distinct Ag specific populations: ASC and non-Ab-secreting memory B cells (B_mem_) [37]. As infections resolve, ASC egress into the circulation and give rise to long-lived plasma cells (PC) residing primarily in bone marrow (BM) [32,33]. ASC emigration from lymphoid follicles is associated with CXCR5 and CCR7 downregulation, resulting in reduced responsiveness to their respective ligands CXCL13 and CCL19/CCL21, which are prominently expressed in lymphoid organs [32,38,39]. By contrast, both CXCR3 and CXCR4 are upregulated during ASC differentiation [27,40], with CXCR4–CXCL12 interactions mediating homing to, and retention within, BM [40,41]. ASC comprise a phenotypically and functionally heterogeneous population of Ag-experienced B cells committed to Ab secretion; they include migration and proliferation-competent, major histocompatibility complex (MHC) class II expressing pre-PC at various differentiation stages, termed plasma blasts (PB), as well as terminally differentiated, sessile, non-dividing PC dedicated to high constitutive Ab secretion [32,33,41,42]. Sessile long-lived PC within the BM maintain protective serum Ab against re-infection [41,42,43,44] with an estimated half-life of anti-viral Ab responses in humans ranging from ~50 to 200 years [45]. The non-Ab-secreting B_mem_ are largely retained in lymphoid organs, but are also found in circulation. Ag re-encountered by B_mem_ mediate rapid transition into ASC by both GC-dependent and independent pathways [42,44,46]. 

**Figure 2:**
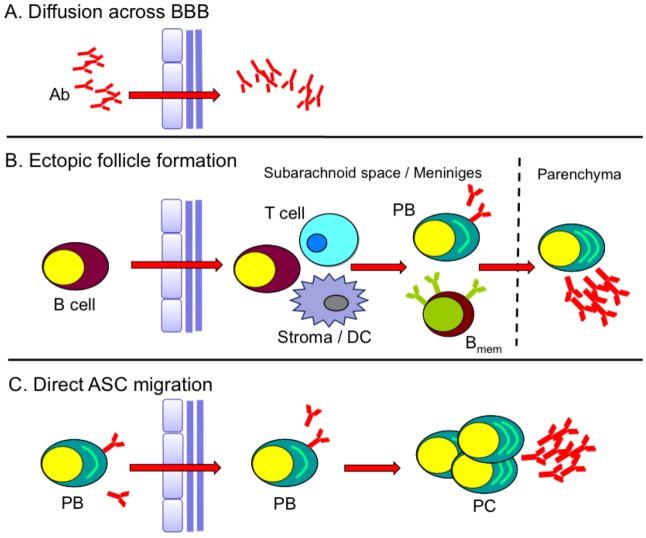
**Models for sustained humoral response in CNS.** (**A**) Direct penetration of serum Ab into the CNS when blood–brain barrier (BBB) integrity is compromised. (**B**) Formation of ectopic follicle-like structures (B cells, T cells, stroma/dendritic cells (DC)) within the subarachnoid space or meninges giving rise to plasma blast (PB) and memory B cells (B_mem_). (**C**) Migration of peripherally activated PB into the CNS that further differentiate into plasma cells (PC).

While the events in lymphoid organs are well documented during immunization or infection of visceral organs, numerous studies have also revealed the presence of ASC in other lymphoid and mucosal tissues, as well as the CNS under various inflammatory conditions [10,11,26,30,32,33]. Moreover, expression of CXCL13 and CCL19/21 within the CNS and other non-lymphoid sites has been associated with formation of ectopic follicle-like structures harboring multiple B cell differentiation phenotypes [47,48,49]. *De novo* Ab production at such sites, as well as upregulation of B cell survival factors [50,51,52], is thought to contribute to clonal B cell expansion and local humoral responses distinct from those occurring systemically. The source of Ab and ASC within the CNS during or following viral encephalitis is largely unknown. Direct penetration of Ab from serum is inefficient due to the blood–brain barrier (BBB). CSF to serum Ab ratios range from ~1:200–1:500 in the healthy CNS [53,54], and are only elevated when BBB integrity is compromised (Figure 2A). Therefore, within the CNS, ASC likely provide a more effective and sustained Ab source. These cells can arise from focal ectopic follicle-like structures within the CNS (Figure 2B) and/or via direct migration of ASC activated in the periphery (Figure 2C). 

## 3. Neurotropic Virus Models

This review focuses on several neurotropic RNA viruses used as murine models of acute encephalitis, demyelinating disease, as well as persistence within the CNS. A common trait of these infections is the failure of T cells alone to provide sterile immunity, and a necessity for sustained local humoral responses to mediate virus control. Murine coronaviruses, also known as mouse hepatitis viruses (MHV), are positive single-stranded, enveloped RNA viruses comprising several strains and isolates that preferentially infect the gastrointestinal and respiratory tract, liver, and CNS. The dual neuro- and hepato-tropic MHV-A59 strain and prominent neurotropic John Howard Muller (JHM) strain, as well as a variety of variant and recombinant viruses, are commonly used to study acute encephalomyelitis and CNS viral persistence associated with demyelination [55,56,57]. Much of the information available on B cells in the CNS discussed herein is derived from studies with the glia-tropic variant of MHV-JHM, designated 2.2v-1 [55]. This virus primarily targets oligodendroglia, and, to a lesser extent, microglia/monocytes in the adult CNS. Neurons are sparsely infected and hepatitis is very rare following intracranial infection, contrasting with the MHV-A59 strain. T cell-dependent control of infectious MHV-JHM occurs within two weeks, independent of humoral immunity. However, persisting viral RNA and pro-inflammatory factors within the CNS demonstrate ongoing, low-level infection despite the continued presence of virus-specific T cells [52,55,58]. While persisting viral RNA levels slowly decline in wild-type (WT) mice, the absence of anti-viral Ab results in the re-emergence of infectious virus after initial viral control [25,59,60]. Viral persistence in a replication-competent form in the face of ongoing immune control is also a hallmark of infection by Sindbis virus (SINV), a prototype alphavirus used to study mechanisms of viral control in neurons [54,61]. SINV is a positive, single-stranded, enveloped RNA virus in the *Togaviridae* family that causes arthritis and rash in humans. Its ability to induce an acute, non-fatal encephalitis in mice provides a useful model for the arthropod-borne alphavirus-associated encephalitic outbreaks in North and South America [54,62,63,64,65]. Like many encephalitic viruses, SINV primarily infects neurons [66,67,68,69,70,71]. In adult mice, infectious virus is cleared within eight days [71], but viral RNA persists in the CNS up to one year after infection [54]. Contrasting SINV, Semliki Forest virus (SFV), another alphavirus, primarily targets neurons and oligodendrocytes [72,73]. In humans, SFV causes fever, persistent headaches, myalgia, arthralgia and rare cases of meningoencephalomyelitis [74,75]. Infection of mice with SFV initiates an acute CNS inflammatory response associated with immune-mediated myelin loss [73]. However, in contrast to SINV and MHV-induced encephalomyelitis, the relative roles of T and B cells in SFV pathogenesis are not as well defined. Rabies virus (RV), a neurotropic, negative-stranded, enveloped RNA virus in the *Rhabdoviridae* family is invariably lethal in humans without intervention. However, post-exposure prophylaxis is only efficacious upon administration of both RV-neutralizing Ab and active immunization [76]. The necessity for the combined immunization regimen in post-exposure prophylaxis corroborates the dual requirement of T and B cell responses for efficient RV control in mice. Nevertheless, RV infection of mice is fatal in the absence of humoral immunity [77], indicating that RV-specific Ab are essential for clearing the virus from the CNS. In contrast to the RNA viruses described above, there is no evidence of persisting viral RNA after clearance of infectious RV. Lastly, Theiler’s murine encephalomyelitis virus (TMEV) is a representative of a positive, single-stranded, non-enveloped RNA virus in the *Picornaviridae* family. Atypical of most other picornavirus infections, strains of TMEV can establish CNS persistence. The ability to persist and cause demyelinating disease in susceptible SJL mice provides a well-studied model of MS [78]. TMEV primarily infects neurons during the acute phase, but establishes persistence in glial cells and macrophages [79,80,81]. However, similar to SFV infection, the role of Ab in anti-viral activity and TMEV persistence, characterized by the presence of infectious virus, remains unresolved. 

## 4. Protective Mechanisms of Humoral Reponses in the CNS

Several lines of evidence highlight the essential role of humoral immunity within the CNS to control infections, either in conjunction with T cells or, subsequently, to peak T cell function. Ab-mediated protection generally correlates with the ability to neutralize virus infectivity. This neutralizing mechanism entails Ab binding to antigenic determinants on virions, thereby preventing attachment and subsequent entry into susceptible cells. Additional viral control mechanisms may involve the deposition of complement components on the virion, resulting in lysis, alterations in coat protein alignment or enhanced phagocytosis. Ab can also impede steps in viral entry or arm natural killer cells and macrophages to induce Ab-dependent cell-mediated cytolysis of virus-infected cells. While non-neutralizing Ab may contribute to the control of acute infection, the mechanism(s) and their ability to regulate viral persistence remains unclear. 

During MHV-A59 infection, loss of viral control during persistence in the absence of humoral responses is unique to the CNS; the virus only recrudesces in the CNS, but not the liver [82]. Moreover, although transfer of immune serum to B cell-deficient mice inhibits recrudescence initially, immune control is lost as Ab wanes [60]. A similar necessity for sustained local Ab secretion in preventing viral recrudescence is evident during SINV infection. Anti-viral Ab transfer to SINV-infected severe combined immunodeficiency mice is only effective in the short term, thereby indicating that Ab controls a persisting reservoir of replication-competent virus [29,83,84]. Passive transfer of virus-specific Ab to RV-infected B cell-deficient mice prior to peak CNS replication reduces—but does not clear—the infection [85], despite access of Ab to the CNS [26]. This therapeutic window coincides with enhanced BBB permeability early during virus CNS invasion and restoration of the BBB prior to viral clearance and peak virus serum Ab levels [86]. Moreover, breach of the BBB is only modest, as indicated by the passage of molecules smaller than 4kDa [87]. BBB integrity during MHV infection is also restored after 14 days (Bergmann CC, unpublished data), potentially contributing to the loss of viral control in the CNS following peripheral Ab transfer. The physiological recovery of BBB integrity following CNS infection highlight a narrow window for serum Ab to contribute to protection once virus infection is established within the CNS. During acute virus encephalitis associated with a breach in BBB integrity, serum Ab may be too low to affect CNS virus control. By contrast, under conditions of minimal sustained infection and inflammation, BBB integrity may be sufficiently restored to exclude circulating protective high affinity neutralizing Ab. 

Passive transfer of virus epitope-specific Ab into humorally impaired mice all reveal viral glycoproteins mediating host cell entry as the crucial targets controlling persistence of enveloped viruses. Thus, only neutralizing Ab directed against the MHV spike (S) protein was efficacious, while Ab blocking fusion or directed against the nucleocapsid (N) protein had no beneficial effects in containing MHV recrudescence [25]. Ab not only acts via neutralization of free virus, but by suppressing viral replication and/or release from persistently infected glia or neurons. This is supported by severely impaired clearance of infectious SINV from neurons in B cell-deficient mice [29]. Furthermore, comparison of anti-SINV monoclonal Ab (mAb) directed at the envelope (E)1 and E2 viral glycoprotein revealed that reactivity to E2 most potently inhibits virus replication by a complement-independent mechanisms [54]. In addition to neutralizing infectious virus, Ab-mediated cross-linking of E2 glycoproteins on the surface of SINV-infected cells prevents virus budding *in vitro* [29,88]. Binding of anti-E2 mAb to infected cells also restores membrane potential, host protein synthesis, and type I interferon (IFN) responsiveness [89,90]. Although the mechanisms underlying suppression of intracellular virus replication remain unclear, these data support the concept that anti-E2 mAb-mediated signal transduction events sustain cell function in the face of ongoing persisting infection. 

The primary target of protective Ab during RV infection is also the glycoprotein responsible for cell entry [91] and neutralization appears to be the primary protective mode [92]. Nevertheless, similar to the SINV E2 glycoprotein, the RV glycoprotein is also expressed on the surface of infected cells, where it may function as a signal-transducing receptor once bound by Ab [93]. Thus, non-cytolytic mechanisms, including inhibition of viral RNA transcription and prevention of cell-to-cell virus spread, may play additional roles in Ab-mediated protection [94]. A similar signaling mechanism has yet to be described for cell surface-expressed MHV S protein engagement by exogenous Ab. Nevertheless, culture of MHV-infected cells in the presence of neutralizing Ab results in persistently infected cells, which only produce progeny virions following fusion with cell lines capable of supporting replication [95]. 

The roles of intrathecal Ab in SFV-induced encephalomyelitis are not as well defined, but studies concur that humoral immunity is required to efficiently control infectious virus [28,96]. While B cell-deficient mice cleared infectious virus from the CNS shortly after 21 days in one study [96], infectious virus remained detectable at eight weeks in another [28]. The role of Ab in SFV-mediated CNS pathology is also contentious. While SVF-induced demyelination is CD8 T cell dependent [97], several studies implicate Ab in contributing to myelin pathology. CSF-derived Ab react with both viral and myelin proteins, indicating that molecular mimicry and/or epitope spreading may exacerbate myelin loss [98]. Consistent with this concept, disease severity is decreased and less white matter vacuolation occurs in SFV-infected B cell-deficient mice compared to WT mice. Moreover, enhanced white matter vacuolation coincides with peak CD19^+^ B cell infiltration into the CNS of WT mice [96]. Pathogenic cross-reactive SFV-induced Ab is also supported by increased ipsilateral turning following microinfusion of serum containing anti-SFV Ab into the rat CNS [99]. By contrast, other studies report no differences in myelin loss comparing SFV-infected WT and B cell-deficient mice [28]. Even a beneficial Ab role is indicated by the ability of anti-SFV E2 Ab to promote remyelination in both SFV-infected mice, as well as mice affected by experimental autoimmune encephalomyelitis [100,101]. 

A potential protective role of TMEV-specific Ab is not well characterized. B cell-deficient mice on the B6 background, resistant to TMEV-induced demyelinating disease, do not exhibit enhanced susceptibility [102], consistent with T cell-mediated viral clearance. Only a combined deficiency in B cells, in addition to the CD8 T cells, results in viral encephalitis and neurological disease [102]. By contrast, viral replication is increased in the CNS of susceptible SJL mice depleted of B cells relative to controls, indicating Ab contributes to TMEV control [103]. While B cells isolated from the CNS of TMEV-infected mice predominantly secrete Ab specific to viral capsid proteins, Ig reactive to several white matter components are also detected in susceptible SJL mice [104]. Furthermore, Ab specific to TMEV capsid protein VP1 cross-reacts with galactocerebroside on myelin and oligodendroglia [105,106]. Although a pathogenic role of Ab in TMEV-infected mice is suggested by the correlation of neurological disability with accumulation of ASC and production of Ab in the CNS, and not serum Ab titers [30], these observations are yet to be confirmed. 

## 5. Kinetics of B Cell CNS Recruitment and Migration Signals

During MHV infection, virus-specific ASC (vASC) initially accumulate in the CNS 10–14 days after infection, peak at three weeks well after the infectious virus is cleared, and slowly decline thereafter [55]. Nevertheless, vASC are detected for at least 90 days after infection, when viral RNA levels are very low. Peak expansion of vASC in lymphoid organs precedes accumulation in the CNS, indicating activation in the periphery [107,108]. All four IgG isotypes are present in the CNS of persistently infected mice with IgG2b > IgG2a > IgG1 > IgG3 [107]. The majority of vASC in the CNS secrete IgG, while smaller fractions produce IgM and IgA [107]. Despite their relatively low frequency, neutralizing IgM ASC alone are sufficient to provide long-term protection from virus-induced demyelination following MHV-A59 infection [109]. ASC accumulating in the CNS within the first two weeks of infection are exclusively specific for the viral S and N proteins; however, they only constitute about 50% of total vASC by day 21 after infection, thus suggesting the emergence of novel ASC specificities during persistence [107]. 

Following SINV infection, extrafollicularly derived SINV-specific IgM^+^ ASC are detected in the CNS by day 3, followed by accumulation and enrichment of SINV-specific IgG^+^ and IgA^+^ ASC [83,110]. SINV-specific IgM in the CNS peaks by day 14, while IgA and IgG are maximal at 1 and 4 months after infection, respectively, at which time IgM has dropped below detection [71]. The early recruitment of IgM^+^ ASC and local IgM production likely cooperate with IFN-γ-secreting CD8 T cells to eliminate infectious virus [61]. Subsequent accumulation of SINV-specific IgG^+^ and IgA^+^ ASC coincides with persisting viral RNA, suggesting these Ig prevent virus reactivation [71]. SINV-specific ASC are detected for at least a year after infection. [83], thereby supporting an essential role for intrathecal Ab production. 

Less is known about the kinetics and isotype specificity of B cells accumulating in the CNS during SFV, RV, and TMEV infections. Following SFV infection, the percentage of CD19^+^ B cells within CNS infiltrating lymphocytes increases from ~20% at day 7, to ~75% at day 21, and significantly drops by day 35 [96]. Furthermore, intrathecal Ab are detected months after infection [111,112]. CD19^+^ B cells are detected in the CNS of RV-infected mice, while mRNAs specific for κ-light chain continue to increase in the CNS weeks after peak viral replication [86]. Moreover, a significantly higher proportion of RV-specific ASC are present in the CNS than in circulation, suggesting vASC are preferentially recruited to, or expand in, the CNS [26]. During TMEV infection, ASC are detected in the CNS five weeks after infection and continue to increase with the predominant isotypes being IgG2a and IgG2b followed by IgA > IgG1 > IgG3 > IgM [104]. Notably, only ~30–40% of ASC in the CNS 2–3 months after TMEV infection are virus specific [30], indicating other yet-to-be-defined specificities, perhaps to self-Ag. Overall, the relevance of IgA, normally associated with mucosal immunity [113] during CNS infections is unclear.

**Figure 3:**
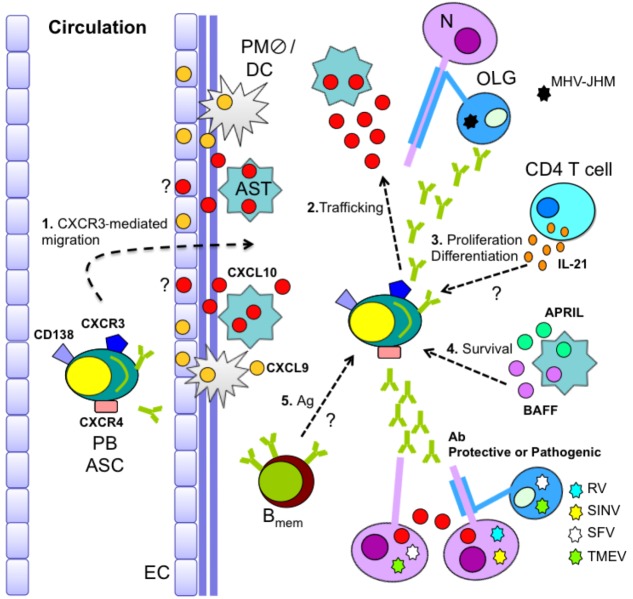
**CNS B cell recruitment/maintenance during glia-tropic MHV infection.** Recruitment of circulating antibody-secreting cells (ASC) into the CNS is CXCR3 dependent (1). While CXCL9 and CXCL10 presentation at the microvasculature may promote initial ASC recruitment, astrocyte-derived CXCL10 mediates ASC entry and parenchymal localization (1 and 2). ASC proliferation/differentiation may be aided by CD4 T cell-derived IL-21 (3). Moreover, sustained elevated expression of astrocyte-derived BAFF and APRIL provides a local niche supporting ASC survival (4). Memory B cell (B_mem_) recruitment and antigen (Ag)-driven differentiation in the inflamed CNS may further contribute to the local ASC population (5). PB = plasma blast; EC = endothelial cell; AST = astrocyte; DC = dendritic cell; PM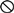 = perivasacular macrophage; N = neuron; OLG = oligoendroglia

The signals promoting and sustaining humoral immunity within the CNS are beginning to be defined. Prominent ASC expansion in cervical lymph nodes prior to CNS migration during MHV-JHM infection is accompanied by their expression of CXCR4 and CXCR3. The absence of CXCR3 specifically impairs ASC recruitment to the CNS, but not BM [27]. CXCR3 thus appears redundant for ASC egress into circulation, but regulates migration to the site of inflammation [27]. CXCR3 expression is also required for accumulation of IgM^+^ ASC in the CNS during MHV-A59 CNS infection [109]. Furthermore, CXCR3 engagement appears to directly mobilize ASC independent of T cells, as neither T cell recruitment nor their anti-viral activity is affected in CXCR3-deficient mice [27,109]. In addition to mediating ASC migration, CXCR3 thus also guides ASC localization within the parenchyma (Figure 3), as indicated by vascular retention of the few ASC detected in the CNS of CXCR3-deficient mice. By contrast, in WT mice, ASC are abundant in the parenchyma and vasculature associated with demyelinated lesions and adjacent white matter [27,108]. Importantly, the inability to maintain control of persisting virus in MHV-JHM-infected CXCR3-deficient mice, although anti-viral serum Ab is not impaired [27], confirms the necessity of intrathecal vASC in preventing viral re-emergence. 

CXCR3-mediated accumulation of ASC is consistent with the sustained expression of the CXCR3 ligands CXCL9, CXCL10, and CXCL11 throughout CNS MHV-JHM infection [52,108]. While CXCL9 expression is strongly dependent on IFN-γ, CXCL10 and CXCL11 are additionally upregulated by IFNα/β and tumor necrosis factor (TNF) [114,115,116]. CXCL10 mRNA is predominantly expressed by astrocytes in the vicinity of viral RNA during MHV-JHM persistence *in vivo* [117]. Analysis of CXCL10 protein localization confirmed astrocytes as the prominent source of CXCL10 [118] (Figure 3). By contrast, CXCL9 protein is confined to the neurovasculature [118] (Figure 3). Despite similar migration capacities of ASC to CXCL9 and CXCL10 *in vitro* [119], ASC accumulation in the CNS was impaired in CXCL10-deficient, but not CXCL9-deficient mice, suggesting CXCL10 is critical to recruit ASC into the CNS during MHV infection [118]. 

The majority of CD19^+^ B cells infiltrating the SINV-infected CNS also express CXCR3 with a smaller proportion expressing CCR5, the receptor for CCL3/CCL5, or CCR7, a receptor for CCL19/CCL2 [120]. CXCL10 and CXCL13, but not CXCL9 or CXCL12, are elevated by day 4 in the CNS of SINV-infected mice [121]; however, the chemokines attracting ASC have not been identified. CXCL13 appears redundant, as infection of CXCL13-deficient mice does not affect CNS accumulation of CD19^+^ B cells [121], leaving CXCL10 as a likely candidate to recruit SINV-specific ASC to the CNS. CXCR3 ligands are also induced in the CNS of SFV, RV and TMEV-infected mice, but their roles in ASC recruitment are yet to be assessed. CXCL9 and CXCL10 mRNA are induced by RV infection and remain elevated as κ-light chain mRNAs accumulate to high levels in the CNS [86,122]. Although RV infection of a microglial cell line directly induces CXCL10 [123], microglia, as a source of CXCL10 *in vivo*, remains to be confirmed. In the TMEV-infected CNS, the biphasic and regionally restricted CXCL10 expression pattern corresponds to the shift in virus replication from the grey to white matter during chronic demyelination [124,125,126]. However neither the source of CXCL10, nor its relevance to humoral CNS immune responses, is characterized. 

It is intriguing that astrocytes are rarely infected by MHV-JHM [127] suggesting their CXCL10 expression is driven by T cell-derived IFN-γ rather than infection itself. CXCL10 is also induced in primary astrocyte cultures infected with TMEV or treated with IFN-γ [128]. These findings support the notion that astrocytes are main CXCL10 producers under inflammatory conditions where IFN-γ levels are high, irrespective of virus tropism. On the other hand, in settings where local IFN-γ production is less prominent, the response of infected cells may regulate ASC recruitment and positioning, especially under conditions of potent pattern recognition receptor and/or IFN-α/β signaling. For example, CXCL10 induction in infected neurons [129] can direct regional migration of T cells and possibly also ASC. Direct comparison of virus variants with distinct tropism, in conjunction with their associated pro-inflammatory responses, may address this question in the future. 

## 6. ASC Differentiation and Survival in the CNS

Ectopic lymphoid follicle-like structures replenishing ASC appear to be absent following viral CNS infections, distinct from chronic CNS autoimmune disorders [130,131,132]. Nevertheless, analysis of both the MHV and SINV models indicate that ASC trafficking to the CNS subsequently differentiate and establish residence. ASC initially recruited to the CNS of MHV and SINV-infected WT mice reveal an early, MHC class II positive, PB phenotype. Subsequent differentiation to a more terminally differentiated sessile PC phenotype is indicated by the gradual loss of MHC class II expression [71,108]. Nevertheless, although PB are the most abundant B cell population late during SINV infection, only few fully differentiated ASC are evident, while B_mem_ cells are readily detectable [71]. The mechanisms driving ongoing differentiation and activation of PB or B_mem_ is unresolved but may reside in CNS chemokines [133], viral Ag, and/or CD4 T cell help. A role of CD4 T cells during both MHV and SINV persistence is implied by sustained expression of IL-10 and IL-21 cytokines [52,120,134] (Figure 3) promoting B cell proliferation/differentiation [135,136,137]. Furthermore, active *in situ* proliferation is supported by Ki-67^+^ and BrdU^+^ B cells in the CNS of persistently SINV-infected mice [120]. 

Irrespective of factors contributing to an increased fraction of differentiated ASC over time, prolonged retention and maintenance of ASC within the CNS is supported by upregulation and sustained expression of BAFF and a proliferating-inducing ligand (APRIL) during infection [52,108,120]. Concomitantly, a portion of B cells infiltrating the SINV-infected CNS express BAFF receptor [120], and transcripts for B cell maturation Ag and transmembrane activator and calcium modulator ligand interactor increase as ASC accumulate during MHV persistence [52]. While elevated BAFF expression is observed in both MHV and SINV infection, APRIL upregulation is only evident during MHV-JHM infection [52,120], suggesting virus-specific regulation of B cell survival factors. Similar to CXCL10, both BAFF and APRIL localize predominantly to astrocytes [52], enforcing their prominent role in recruitment, as well as their maintenance of ASC during MHV-JHM infection (Figure 3). 

## 7. Summary and Future Perspectives

The inflamed CNS provides a supportive microenvironment for intrathecal Ab production. In fact, the milieu within the CNS associated with RNA viral persistence is characterized by long-term retention of ASC, which appear essential in preventing viral recrudescence. Increasing evidence demonstrates that virus-induced humoral responses within the CNS are highly dynamic with early recruitment of naive B cells, as well as IgM^+^ and IgA^+^ PB, which are gradually replaced by IgG^+^ PB with time. Ongoing local B cell differentiation is further supported by a more differentiated ASC phenotype resembling the long-lived PC found in the BM. However, unlike chronic autoimmune inflammation or neuroborreliosis [10,131,132], there is little evidence for ectopic lymphoid-like follicle formation as a consequence of viral encephalitis or persistence. Rather, ASC appear directly recruited from peripheral lymphoid organs, with CXCR3-mediated signaling providing essential migration cues. The source of CXCR3 ligands within the CNS appears dependent on viral tropism, as well as induction of IFN-α/β and strength of pro-inflammatory signals such as TNF and IFN-γ. Astrocytes may be strategically relevant for ASC accumulation and survival based on their integral role in maintaining the glia limitans component of the BBB and bystander capacity to produce the CXCR3 ligand CXCL10, as well as the ASC survival factors BAFF and APRIL. Whether activated astrocytes play additional roles in promoting B cell differentiation via chemokine or cytokine expression remains to be investigated. Similarly, additional roles of dendritic type cells, viral Ag, and CD4 T cells in driving B cell differentiation locally remain to be explored. Overall, the reliance on local ASC for prolonged protective Ab output, under conditions in which the BBB is relatively intact, may be critical in providing sustained immune control of numerous neurotropic infections. The highly tenuous balance in immune control of persisting viruses is revealed by targeted intervention blocking lymphocyte trafficking in MS patients [23,138]. Future focus on the factors regulating B cell differentiation and/or CNS entry, such as IL-21 or CXCL10, may lead to more efficacious strategies to enhance protective humoral immunity during viral encephalitis or impede accumulation of detrimental B cells.
